# Phenotypic and genetic integration of personality and growth under competition in the sheepshead swordtail, *Xiphophorus birchmanni*

**DOI:** 10.1111/evo.13398

**Published:** 2017-11-30

**Authors:** Kay Boulton, Craig A. Walling, Andrew J. Grimmer, Gil G. Rosenthal, Alastair J. Wilson

**Affiliations:** ^1^ The Roslin Institute and Royal (Dick) School of Veterinary Studies University of Edinburgh Easter Bush Midlothian EH25 9RG United Kingdom; ^2^ Institute of Evolutionary Biology University of Edinburgh Ashworth Laboratories Edinburgh EH9 3FL United Kingdom; ^3^ Marine Biology and Ecology Research Centre, School of Biology and Marine Sciences Plymouth University Drake Circus Plymouth Devon PL48AA United Kingdom; ^4^ Department of Biology Texas A&M University 3258 TAMU College Station Texas 77843; ^5^ Centro de Investigaciones Científicas de las Huastecas “Aguazarca,” Calnali Hidalgo Mexico; ^6^ Centre for Ecology and Conservation, Biosciences, College of Life and Environmental Sciences University of Exeter Cornwall Campus Penryn Cornwall TR10 9EZ United Kingdom

**Keywords:** competition, G matrix, indirect genetic effects, quantitative genetics, Xiphophorus

## Abstract

Competition for resources including food, physical space, and potential mates is a fundamental ecological process shaping variation in individual phenotype and fitness. The evolution of competitive ability, in particular social dominance, depends on genetic (co)variation among traits causal (e.g., behavior) or consequent (e.g., growth) to competitive outcomes. If dominance is heritable, it will generate both direct and indirect genetic effects (IGE) on resource‐dependent traits. The latter are expected to impose evolutionary constraint because winners necessarily gain resources at the expense of losers. We varied competition in a population of sheepshead swordtails, *Xiphophorus birchmanni*, to investigate effects on behavior, size, growth, and survival. We then applied quantitative genetic analyses to determine (i) whether competition leads to phenotypic and/or genetic integration of behavior with life history and (ii) the potential for IGE to constrain life history evolution. Size, growth, and survival were reduced at high competition. Male dominance was repeatable and dominant individuals show higher growth and survival. Additive genetic contributions to phenotypic covariance were significant, with the **G** matrix largely recapitulating phenotypic relationships. Social dominance has a low but significant heritability and is strongly genetically correlated with size and growth. Assuming causal dependence of growth on dominance, hidden IGE will therefore reduce evolutionary potential.

An individual's phenotype is determined by its genotype and the environment it experiences throughout life. Competition with conspecifics for resources (e.g., food, space, mating opportunities) is one important environmental factor known to have large effects on phenotypic traits including growth (Ruzzante and Doyle 1991) and life‐history traits (e.g., maturation, fecundity, longevity). Importantly, by producing winners and losers, competition generates variation in resource‐dependent traits and ultimately in fitness. Since winners increase their (relative) fitness at the expense of losers (Brockelman 1975), those traits contributing to competitive ability are also expected to be under strong selection. If so, then the evolutionary consequences of this selection will depend on the genetic covariance structure between traits causal and consequent to social dominance (Wilson 2014). In the particular case that dominance itself is heritable, this genetic covariance will include contributions from indirect genetic effects (IGE; Bijma and Wade 2008) that can constrain adaptation of resource‐dependent traits (Wilson 2014). Here, we use a quantitative genetic approach to characterise the genetic basis of social dominance in a population of the poeciliid fish *Xiphophorus birchmanni* and explore the extent that genetic and environmental effects, notably including the degree of competition itself, shape the multivariate phenotype. Our goals are to assess the extent to which competition leads to phenotypic and/or genetic integration of behavioral and life‐history traits, and to evaluate the potential for constraining IGE on the latter.

In animals, intraspecific competition takes different forms and occurs across many different social contexts–from pure scramble competition leading to density dependence (Hassel 1975), to dyadic contests that can escalate to become an important source of mortality (e.g., Liker and Szekely 2005). From an ecological perspective, competition reduces mean (absolute) fitness with consequences for regulation and determination of population demography (Schoener 1983; Sih et al. 1985; Chase et al. 2002). However, in evolutionary terms perhaps the most important role of competition is as a mechanism that generates among‐individual variation in both phenotypes and fitness. Within populations, individuals can vary in competitive ability, or social *dominance*, defined here as an individual's repeatable tendency to win or hold resources under competition (Wilson et al. 2011a). Note that this definition is simply phenomenological, and certainly does not imply dominance is determined solely by “intrinsic” factors. Indeed the converse is true; winning resources usually depends on both focal phenotype and the particular social context or environment provided by competitors.

More competitive phenotypes should generally be favored by selection, and this has implications for the evolution of traits both causal and consequent to competitive outcomes. Although social dominance is not necessarily without costs (e.g., Wong and Kokko 2005; Bell et al. 2012) overall, dominant individuals win resources and thus ultimately gain relative fitness at the expense of subordinates. This in turn allows increased investment in, for instance, growth, earlier maturation, or reproductive effort (Bernstein 1976; Huntingford et al. 1990; Fox et al. 1997). Where fitness is tightly linked to competitive outcome, traits determining dominance might be under strong directional selection (Kruuk et al. 2002; Benson and Basolo 2006; Prenter et al. 2008). Simple evolutionary theory predicts that, all else being equal, this should erode genetic variance (Fisher 1958). If so, then at equilibrium phenotypic variation in traits determining dominance (and so dominance itself) will largely be due to environmental effects (Kruuk et al. 2002). However, directional selection on contest outcome can also generate disruptive selection on, and so maintenance of variation in, quantitative traits that mediate competitive outcomes (e.g., Abrams et al. 2008).

Although the limited number of studies conducted to date have generally found relatively low heritabilities for measures of social dominance (Wilson et al. 2011b; Sartori and Montavani 2012), this may reflect high environmental variance rather than an absence of genetic effects. For instance, much of our understanding of dominance comes from dyadic animal contest studies where winning is often causally dependent on heritable aspects of morphology such as body size and/or weapons (e.g., horns, Preston et al. 2003). More recently, there has been growing recognition that social dominance can also depend on an individual's (repeatable) behavioral phenotype, or personality (Reale et al. 2010). Evidence is now accumulating that personality traits linked to competition, such as aggression and boldness (loosely defined as a willingness to take risks) are also heritable (e.g., Drent et al. 2003; Sinn et al. 2006; Ariyomo et al. 2013). Integration of multiple behavioral and morphological traits could result in alternative “strategies” for success in competition having equal fitness and/or being maintained by frequency‐dependent selection. This may contribute to the maintenance of genetic variance in traits causal to dominance (as we broadly define it) and could occur if, for example, large aggressive individuals succeed in contest competition, but smaller and bolder (or more exploratory) individuals do well in more scramble‐like competition. Such a scenario could potentially explain the maintenance of sneaker male morphs in systems with male–male competition for females (e.g., Ryan et al. 1992). However, across taxa the emerging—albeit certainly imperfect—pattern is one of positive covariance between boldness and aggression (e.g., Johnson and Sih 2005; Pintor et al. 2008; Ariyomo and Watt 2012). Furthermore, both these personality traits are commonly positively associated with social dominance, resource‐dependent life‐history traits and fitness measures (e.g., Biro and Stamps 2008; Ariyomo and Watt 2012; Rudin and Briffa 2012).

Regardless of the relative importance of morphological and behavioral traits, it seems likely that social dominance will often be determined by genetically variable components of phenotype and so can be viewed as a heritable trait in its own right. If so, this has important implications for our understanding of life‐history evolution. This is because genes that increase dominance will allow individuals to succeed in competition, gain more resources, and so invest more in all resource‐dependent life‐history traits. Genetic variance in dominance will therefore be a source not only of heritable variation in downstream traits, but also of positive genetic covariance (defined with respect to fitness consequences) between traits subject to resource‐dependent trade‐offs (Wilson 2014). Positive genetic correlations are a common empirical finding in natural populations (Kruuk et al. 2008) and seemingly pose a challenge to the view that trade‐offs, expected to manifest as negative genetic correlations, ubiquitously impose evolutionary constraint.

However, genetic variance in dominance, or competitive ability, is also expected to generate indirect genetic effects (IGE). IGE occur when the phenotype of one individual is causally dependent on the genotype of another, and are inevitable in the case that dominance is genetically variable. IGEs can have important implications for predicting evolutionary responses, and in particular are predicted to dampen the response of resource‐dependent traits when they arise from competitive interactions. This is because selection on these trait(s) is expected to result in the correlated evolution of a more competitive social environment that offsets the expected phenotypic change (Hadfield 2010; Wolf et al. 1998; Bijma and Wade 2008; Wilson et al. 2011b). Consequently, while genetic (co)variance is the raw material for adaptive evolution, understanding the extent that it is independent of social competition (and thus constraining IGE) may be a prerequisite for predicting selection responses (Hadfield et al. 2011; Wilson 2014).

Here, we tested the genetic basis of dominance, and characterized both genetic and environmental contributions to covariance in and between dominance, personality, size and growth, and survivorship in a laboratory population of the sheepshead swordtail, *Xiphophorus birchmanni*. Swordtails have been widely used in studies of social dominance (e.g., see Earley 2006 for an historical review; Walling et al. 2007; Boulton et al. 2012), while previous work on this particular population has found evidence of stable personality traits including aggressiveness (Wilson et al. 2013) and boldness (Boulton et al. 2014). In adult males, aggressiveness has been shown to be a better predictor of dyadic contest outcome than body size (Wilson et al. 2013). The ability to win food in dyadic contests is repeatable in adults of both sexes, while dominant individuals (i.e., those that consistently win) tend to gain weight at the expense of subordinates (Wilson et al. 2013).

In our experiment we use density manipulations to increase the expected intensity of competition in the sheepshead swordtail. After testing for competition effects on mean growth, personality, and survival, we estimate the among‐individual and genetic covariance structures between traits related to social dominance. We manipulate competition by subjecting a captive bred generation of fish to contrasting low (L) and high (H) competition treatments in both early and later life. We hypothesise that high competition (i.e., high density), particularly if experienced in early life, will reduce growth rates and negatively impact fitness components (e.g., survival). Having shown direct effects of competition on phenotypic expression, we use a multivariate modeling approach to estimate the relationships among traits at the individual and additive genetic levels. We predict that personality differences will predict social dominance with bolder individuals tending to be dominant. We also expect that social dominance will positively predict growth and survival. If heritable variation for dominance is present, then similar correlation structure is expected at the (direct additive) genetic level. Importantly, if this is this case the (direct) genetic (co)variance structure among resource‐dependent traits (**G**) will be insufficient to predict selection responses. More specifically, **G** will give an upwardly biased expectation of the potential for adaptive evolution because it does not account for IGE that will necessarily arise if dominance is heritable.

## Materials and Methods

Data analyzed here were from a broader study (Boulton et al. 2014; Boulton et al. 2016). Previously described methods are thus kept accordingly brief. Behavioral data from open field trials (OFT) have been previously published (Boulton et al. 2014) but not subjected to genetic analyses.

### HUSBANDRY AND DENSITY TREATMENTS

Briefly, one hundred adult fish (60 female and 40 male *Xiphophorus birchmanni*) were caught from the Río Coacuilco, in the municipality of San Felipe Orizatlán, Hidalgo, Mexico, and imported to the United Kingdom. Between August 2010 and May 2011, these fish were crossed to produce 384 offspring comprising 61 families nested within a half‐sib structure (with 19 male and 32 female parents represented). Families of ≤8 individuals were raised in one half of a brood tank (37 × 37 × 22 cm capacity partitioned into two equal volumes with a mesh divider). Full‐sib families of >8 individuals were divided equally across two partitions (in different tanks). Groups of six brood tanks (and eight experimental housing tanks; described below), henceforth referred to as *stacks* shared a single recirculating water supply. Offspring were fed twice daily (fresh brine shrimp nauplii and a mix comprising equal quantities of crushed spirulina and brine shrimp flake). At an average age of 16 weeks (range 12–27) and length 27 mm (20–35), fish were tagged below the dorsal fin with coloured elastomer and assigned to mixed family groups (*n* = 8). Each group was subject to one of two initial density treatments; low (L) density groups were housed in a full tank (37 × 37 × 22 cm), while high (H) density groups in a partitioned tank (i.e., half the volume). Six stacks were set up sequentially (each comprising four L and four H groups) as sufficient fish (64 per stack) reached sufficient size to enable individual identification by use of elastomer tags. Sex ratio was not controlled as external sexing of juveniles is not possible. All groups received the same food ration (commercial flake and frozen tropical fish food) twice daily. After 28 weeks (subsequently referred to as part 1), density treatments were reversed for four randomly chosen groups within each stack. All groups were maintained for a further 22 weeks (part 2 of the study). Thus within each stack, four density regimes were experienced (LL, LH, HL, HH), with two groups per regime. Natural mortality over the course of the experiment resulted in some variation in group size (initially eight) through time, although survival was high (368 of 384) over the first density treatment period (i.e., part 1 of the study).

### PHENOTYPING METHODS

Behavioral data were collected on boldness and dominance. Boldness was determined using open field trials (OFT) described fully in Boulton et al. (2014). Individuals were subject to a total of four OFT: two in part 1 (weeks 13 and 21) and two in part 2 (weeks 33 and 41). At each trial a fish was introduced to a 45 × 25 × 25 glass tank filled to a depth of 8 cm with room temperature water (22°C). After 30 s acclimation, a five minute observation period was filmed and a suite of traits putatively indicative of boldness extracted from the video using the Biobserve Viewer tracking software. Our previous analysis shows the among‐individual (i.e., repeatable) component of multivariate variation is dominated by a single major axis of variance, broadly matching expectations of a shy‐bold continuum (Boulton et al. 2014). Thus, here we selected a single trait, activity (percentage time in trial spent moving at >1.5 cm.s^−1^) for use as a proxy for boldness.

Social dominance was assayed for males only using in‐tank observations (ITO). Behavior of each male in each group was recorded for five minutes, at up to five occasions during the experiment: two at the initial density treatment during part 1 of the study (weeks 18 and 25), and three at the final density treatments during part 2 of the study (30, 38, 44 weeks). Remaining males at the end of part 2 were pooled with others from their stack in a large tank (45 × 120 × 30 cm) containing previously unencountered stock females and observed on a minimum of five further occasions (consecutive days where possible). Within groups, focal males (identifiable from natural markings and elastomer tags) were watched sequentially in a haphazard order by a recorder seated in front of the tanks in full view of the fish. Fish were accustomed to researcher presence and our judgement was that this did not impact behavior.

While we acknowledge that aggression (actual, threat, or signal of attack, Hand 1986, Francis 1988) and dominance are not equivalent, the former is often used to assert the latter (Bernstein 1976). Here, we have previously shown that aggressive behaviors predict feeding dominance among male *X. birchmanni* (Wilson et al. 2013) while male dominance is known to determine access to females in swordtails generally (Magellan and Kaiser 2010). For each five minute observation period, a within‐group dominance score was therefore assigned to each focal male as the total number of aggressive actions toward other males (attacks, dorsal fin displays, chases), plus the number of courting attempts (displaying to female, shepherding away from other males), minus the number of submissions (retreating or fleeing from another male) and aggressive acts received (see Wilson et al. 2013 for further description of these behaviors).

Finally, standard length (SL) and live mass (WT) were measured at tagging (measure 1) and subsequent four‐weekly intervals. Up to 13 measures were made on each fish (with measure eight corresponding to the end of part 1, and measure 13 the end of part 2). We also recorded longevity as the age at death in days (regardless of whether death was natural or by euthanasia), and right‐censored to age at the end of part 2 of the experiment (for fish alive at measure 13).

### STATISTICAL ANALYSES

Following graphical exploration of the raw data, density treatment, and genetic effects on behavior, size, and growth were tested using (univariate) linear‐mixed effect models, including pedigree‐based animal models (Wilson et al. 2010) fitted by restricted maximum likelihood (REML) in ASReml‐R. Conditional *F*‐tests were used for inference on fixed effects in the univariate models, with sequential dropping of nonsignificant terms (but retaining main effects in the presence of significant interactions). Starting fixed effects were included to control for potentially confounding effects, and to test for density treatment effects. Inference on random effects was by likelihood ratio test (LRT). We follow Self and Liang (1987) by assuming the test statistic is distributed as a 50:50 mix χ^2^_0_ and χ^2^_1_ (denoted χ^2^_0,1_) for tests of a single variance component. For comparing models differing in more parameters (e.g., random regressions and multivariate models described below) we adopted the more conservative strategy of setting DF equal to the number of additional covariance components in the more complex model.

### DETERMINATION OF FIXED EFFECTS IN UNIVARIATE MODELS

To test density treatment effects two‐level factors were defined corresponding to early life density (*ELD*; L vs H in part 1) and late life density (*LLD*, L vs H in part 2). Thus *ELD*:*LLD* defines a factor specifying the full regime (LL, LH, HL HH). Since *LLD* treatment cannot influence phenotypic observations made during part 1 of the experiment, effects were fitted only to part‐specific data (denoted by subscript) where appropriate. *ELD_part1_*, *LLD_part2_*, and *ELD:LLD_part2_* were therefore fitted for *activity*, *dominance score*, *SL*, and *WT*.

All starting models included fixed effects of *stack* (a six‐level factor), *sex* (except *dominance score* since male‐limited), *sex ratio* (*SR*), and *group size* (*GS*). *Group size* (*GS*) and *sex ratio* (*SR*) experienced were defined for each individual (*i*) rather than each group. *GS* was defined as the geometric mean number of fish in *i*’s group, averaged across months up to and including the observation, and included to control for effects of mortality (reducing group size from the starting n = 8). *SR* was similarly defined as the geometric mean (across previous months) of the proportion of i's tank mates that are mature males (see Boulton et al 2016). Both variables were (arithmetic) mean‐centered across all individuals to aid model interpretation. For behaviours, additional fixed effects included: *trial* (factor, the number of previous assays experiences); *order* (zero‐centred covariate, indicating the trialling sequence of individuals tested on a day); and *observation type* (within‐group during main experiment versus in larger tank after) for *dominance score*. For size traits (*SL* and *WT*) we included *Measure* (a factor with 13 levels) and *Measure:Sex*, allowing sex‐specific average growth patterns over the 13 months. A linear effect of absolute *Age* (zero‐centred) was also included to account for variation in age among fish entering the experiment.

### RANDOM EFFECT SPECIFICATION IN UNIVARIATE MODELS

Random additive genetic and permanent environment effects were fitted using a standard repeat measures animal model (Wilson et al 2010). For *SL* and *WT* this model was extended to include 1st order (linear) random regressions on age (zero centred on the mean age of 294 days) for both additive and permanent environment effects (following e.g., Wilson et al. 2006). This partitions each individual's genetic deviation from the mean trajectory of size over age into a random intercept, and a random slope. Variance in the former represents genetic variance in size (at average age), the latter genetic variance in growth. Environmental deviations from the mean size are treated analogously to partition the nongenetic component of among‐individual variation in size (at average age) and growth. Repeatability (R) was estimated as the ratio of among‐individual variance (V_I_) to phenotypic variance (V_P_) conditional on fixed effects using a simple repeat measures mixed model containing *identity* and (for *SL* and *WT* only), *identity* x *age* as random effects. Narrow sense heritabilities, h^2^ were estimated from the animal models as the ratio of V_A_ to V_P_. We did not generate R or h^2^ estimates for *Growth* (as inferred from either *SL* or *WT*) because among‐individual and additive genetic variances in reaction norm slopes are estimated from the random regression models but residual variances are not.

### ANALYSIS OF SURVIVAL DATA

Right censored age of death data were analyzed using a proportional hazards regression model implemented by coxph in the R library survival (Therneau and Grambsch 2000; Therneau 2015). Predictors of *Stack*, *Sex*, *GS_i_, SR_i,_* and *ELD:LLD* were included. Note that mortality occurred almost exclusively in part 2 so separate effects of ELD and LLD were not modelled. A small number of fish that died with indeterminate sex were excluded. Heritable variation in *survival*, defined as zero (dead before measure 13) or one was also tested for using a univariate animal model and the same fixed predictors with an addition linear effect of age at Measure 1. We assumed a Gaussian error structure to obtain an estimate of heritability on the observed (i.e., 0/1) data scale, but note that statistical inferences from this model parameters should be treated caution as a consequence.

### MULTIVARIATE MODELS TO ESTIMATE ID, G AND SELECTION THROUGH *LONGEVITY*

Multivariate‐mixed models were then used to estimate **ID,** the among‐individual phenotypic variance‐covariance matrix, and the additive genetic matrix **G**. These were fitted in the standalone implementation of ASReml (v4) assuming Gaussian residuals. The 6 × 6 **ID** matrix was first estimated among the set of observed traits (*activity*, *dominance score, SL*_,_ *WT*) and the two growth traits (*Growth_SL_* and *Growth_WT_* modeled using random regressions). Observed traits were scaled to standard deviation units to facilitate convergence and fixed effects included on each trait as determined from univariate analyses. A random effect of individual identity was included on all traits. This model was compared to one where **ID** was constrained to be a diagonal matrix (i.e., all covariance elements equal to zero) as an overall test of among‐trait covariance. The original model was then extended to include *survival* (0, 1) as an additional response variable. *Survival* is observed once only, such that V_I_ and V_R_ are not separable and the latter was therefore fixed to zero. This partitions all variance in survival (conditional on fixed effects) to the extended **ID** structure. Observed *survival* was divided by the mean to convert to relative fitness. The covariance estimates between each trait and relative fitness in **ID** can then be interpreted as the ordinary selection differentials of quantitative genetic theory (Falconer and Mackay 1996) contained within a vector **S**. We compared this model's fit to one where all elements of **S** are fixed to zero as a global test for selection.

The above steps were repeated using male and female data separately to qualitatively check whether pooling sexes for multivariate genetic analysis was sensible, and determined whether selection was similar across sexes. **ID_male_** and **ID_female_** were broadly similar (apart from necessary exclusion of *dominance score* in females; see results) so power was maximised by estimating **G** from a pooled‐sex multivariate animal model. We note that if G x *Sex* interactions occur, the resultant estimate of **G** can be viewed as an average of sex‐specific matrices. Trait‐specific fixed effects were included as before, with random additive genetic and permanent environment effects on all traits. For *SL* and *WT* first‐order random regressions of *age* were used. The full model fit was compared to one with a diagonal **G** matrix assumed as a global test for genetic covariance among traits and individual COV_A_ estimates were scaled to genetic correlations (r_G_) to facilitate interpretation. Given a lack of V_A_ in the univariate analysis, we did not expand this analysis to include (relative) *survival*.

## Results

### DATA STRUCTURE AND FIXED‐EFFECTS ON TRAITS

**Figure 1:**
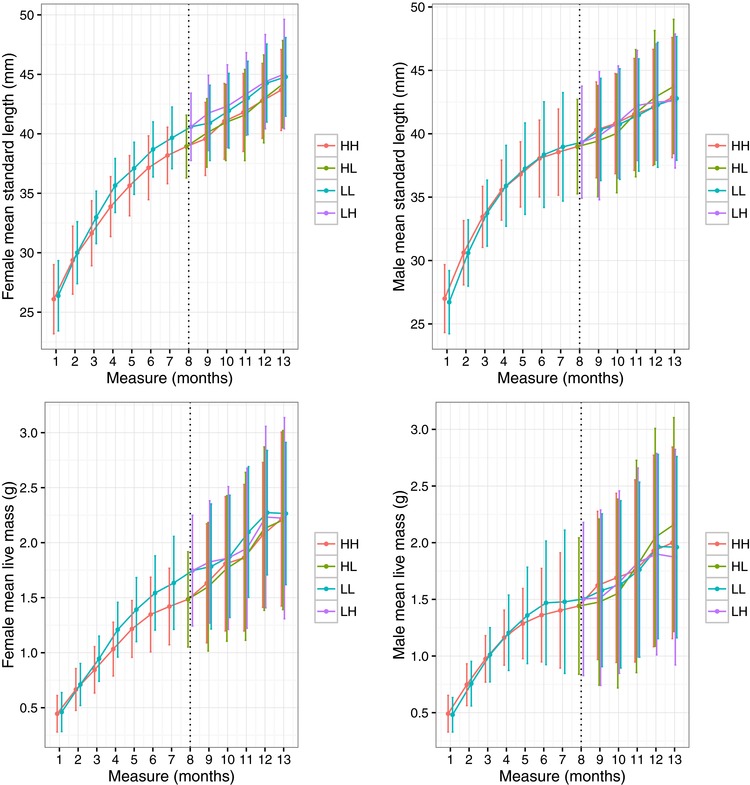
Average growth trajectories showing mean standard length (A – females, B ‐ males) and live mass (C – females, D – males) by month from the start of the experiment for fish in each density treatment regime. Bars indicate standard deviations and dashed line indicates the point of treatment switch.

The final dataset included 384 individuals (222 males, 151 females, 11 fish with undetermined sex at time of death or end of data collection period); 4175 age‐specific measures of size (*SL* and *WT*); 1235 observations of *activity* in OFT; and 1385 observations of male *dominance score*. Visual comparison suggests mean growth trajectories are similar across sexes (for *SL* and *WT*; Fig. 1), although standard deviations for size at each age are uniformly larger in males (Fig. 1A vs 1B, 1C vs 1D). On average, growth continued across the study timeline in all density treatments, although the comparatively constant rate of absolute growth in part 2 masks a decline in relative growth rates after maturation in both sexes (see Fig. S1).

Effects of density treatment were not obvious from visual inspection of behavioral data (not shown), while univariate models confirmed no significant density treatment effects on *activity* or *dominance score* (Table 1; see also Table S1 for coefficient estimates). In contrast, high part 1 density reduces age‐specific *SL* and *WT* (especially in females; Fig. 1A and C). Fish experiencing low density in early life were bigger in part 1, both in terms of *SL* (*ELD_part1_* L coefficient (SE) = 0.635 (0.113), F_1,2583.6_ = 31.5, *P* < 0.001) and *WT* (*ELD_part1_* L coefficient (SE) = 0.050 (0.020), F_1,2583.6_ = 31.5, *P* < 0.001). Significant effects of *LLD_part2_* and (*LLD:ELD)_Part2_* on *WT* were also detected (Table 1). Collapsing these terms into a four level factor defining possible treatment combinations on *WT_part2_* yields effect size estimates (relative to expected mean for HH fish) of LL –0.038 (0.039) , LH –0.165 (0.039), HL 0.024 (0.033). Thus, the significant effect of LLD on *WT*_part 2_ is driven largely by a negative impact of switching from L to H at the end of part 1.

**Table 1:** Fixed effects retained for each trait showing results of conditional Wald F tests from univariate animal models including additive genetic (all traits) and permanent environment (all traits except longevity) effects as random

Trait	Effect	*F*	DF	*P*
*Activity*	Intercept	1332	1, 12.1	<0.001
	Stack	7.80	5, 188.3	<0.001
	Sex	7.51	1, 345.8	0.006
	Trial	131	3, 921.6	<0.001
	Order	10.4	1, 1203.9	0.001
*Dominance score*	Intercept	12.8	1, 11.6	0.004
	Trial	3.26	15, 1220.4	<0.001
*Standard length*	Intercept	4196	1, 1.5	0.002
	Age	22.4	1, 494.3	<0.001
	Measure	462	12, 3335.5	<0.001
	Stack	2.43	5, 343.6	0.035
	Sex	0.362	1, 361.4	0.548
	GS	7.22	1, 3824.1	0.007
	Measure:Sex	13.9	12, 3384.3	<0.001
	ELD_Part1_	31.5	1, 2583.6	<0.001
*Weight*	Intercept	486	1, 6.7	<0.001
	Age	19.7	1, 279.1	<0.001
	Measure	169	12, 3123.3	<0.001
	Stack	4.39	5, 330.1	0.001
	Sex	0.674	1, 359	0.412
	Measure:Sex	13.4	12, 3477	<0.001
	ELD_Part1_	47.1	1, 837.1	<0.001
	LLD_Part2_	11.8	1, 3782.9	0.001
	(ELD:LLD)_Part2_	8.69	2, 2621.5	<0.001

Based on significance in univariate models, some additional fixed effects were retained in variance component and multivariate analyses that are not directly relevant to current hypotheses, therefore we describe them only briefly here (but see Table 1 and Table S1 for full presentation). Specifically *sex* effects are present on mean size (*SL* and *WT*) and average growth trajectory (manifest as *Measure:Sex* effects) and *GS* effects positively influenced *SL*. *SR* was not a significant predictor of any trait. Among‐*stack* differences were present for all traits except *dominance score*. For *activity*, significant *trial* effects were driven by greater mean *activity* at trials 2–4 relative to trial 1, while a negative effect of *order* was also present. These were already known from prior analysis of OFT data (Boulton et al 2014). *Trial* also significantly influenced *dominance score*, with higher scores seen in the later observations made after mixing fish across units. This is consistent with an expected increase in agonistic interactions among individuals that are unfamiliar with each other (see e.g., Wong and Balshine 2011).

### REPEATABILITIES, HERITABILITIES, AND (GENETIC) VARIANCE IN GROWTH

Univariate models provided statistical support for among‐individual variance (conditional on fixed effects), underpinned by genetic effects for all traits (but not survival as noted above). Testing random effects in univariate models confirmed significant repeatability of *activity* (*R* = 0.260 (0.034)) and male *dominance score* (*R* = 0.242 (0.032); Table 2). Behavioral heritabilities were low (h^2^_activity_ = 0.093 (0.056), h^2^_Dominance_ = 0.066 (0.052)) but V_A_ estimates significant at *P* < 0.05 (Table 2). Size varied among individuals (*SL* and *WT*) and first‐order random regression models yielded significantly better fits than simple repeated measures models. Thus there is among‐individual variance in both size and growth (the latter being I x *age* for size; Table 2). This was mirrored at the genetic level with V_A_ and G x *age* interaction statistically supported for *SL* and *WT* (Table 2). Noting that ID x *age* and G x *age* imply age‐dependence of V_I_ and V_A_, respectively, we estimate *R*_SL_ = 0.908 (0.008) and *R*_WT_ = 0.839 (0.011) at 294 days (the mean observed age in the dataset). At this age h^2^_SL_ = 0.247 (0.106) while h^2^_WT_ is lower (though not significantly so) at 0.144 (0.076).

**Table 2:** Repeatability (*R*) and heritability (h^2^) estimates for all traits and survival

			V_I_		V_A_		ID x Age		G x Age	
Trait	*R* (SE)	h^2^ (SE)	χ^2^_0,1_	*P*	χ^2^_0,1_	*P*	χ^2^_2_	*P*	χ^2^_2_	*P*
*Activity*	0.260 (0.034)	0.093 (0.056)	83.3	<0.001	4.97	0.013	—	—	—	—
*Dominance score*	0.242 (0.032)	0.066 (0.052)	180	<0.001	3.66	0.028	—	—	—	—
*Standard length* 1	0.908 (0.008)	0.247 (0.106)	3370	<0.001	19.61	<0.001	3387	<0.001	17.6	<0.001
*Weight* 1	0.839 (0.011)	0.144 (0.076)	2030	<0.001	14.14	<0.001	3587	<0.001	11.2	0.004
*Survival*	—	0.016 (0.015)	—	—	1.93	0.082	—	—	—	—

^1^Since random regression models are used *R* and h^2^ estimates here are for size at mean observed age of fish in the study (= 294 days).

Estimates are from univariate models and standard errors in parentheses. Also shown are likelihood ratio tests of among‐individual variance (V_I_), additive genetic variance (V_A_) and, for size traits only, among‐individual (ID x Age) and additive genetic (G x Age) variance in growth.

### SURVIVAL ANALYSIS

**Figure 2:**
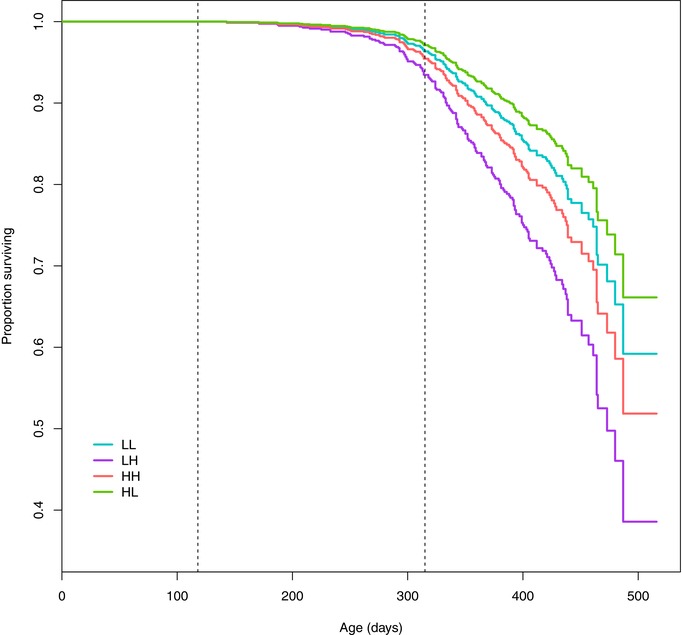
Predicted survival curves by total density treatment. From left to right, the dashed vertical lines denote mean age at start of the experiment, and mean age at measure 8 (end of part1).

Two hundred fish (52.8%) remained alive at the end of part 2 (measure 13). Observed survival to measure 13 was higher in females (62.0% vs 46.6% in males) with treatment‐specific rates (sexes combined) of LL = 59.1%, LH = 46.9%, HL = 55.9%, and HH = 49.5%. Testing of the treatment effects in the proportional hazards regression predicts that, conditional on other model effects, survival is lower in LH and HH and higher in HL relative to LL (the reference treatment level (Fig. 2)). However, only in LH is the difference from LL statistically significant (Table 3). Thus, experiencing low density in early life and then being switched to high density has a negative effect on survival. In addition there were significant effects of *Stack*, *SR*, and *GS* (with the hazard for a focal individual increased in more male‐biased and larger groups; Table 3). The animal model of survival yielded a small nonsignificant estimated for survival on the observed 0/1 scale of h^2^ = 0.016 (0.015).

**Table 3:** Results of survival analysis using proportional hazards regression model

Predictor	Coefficient	SE	Exp (coefficient)	*z*	*P*
Stack (A)	NA				
Stack (B)	1.087	0.274	2.965	3.971	<0.001
Stack (D)	0.270	0.271	1.310	0.995	0.320
Stack (E)	0.808	0.281	2.244	2.879	0.004
Stack (F)	0.120	0.305	1.127	0.392	0.695
Stack (G)	–0.185	0.304	0.831	−0.607	0.544
Sex (Female)	NA				
Sex (male)	0.204	0.183	1.226	1.114	0.265
GS	1.720	0.174	5.584	9.885	<0.001
SR	0.829	0.388	2.290	2.134	0.033
Treatment (LL)	NA				
Treatment (LH)	0.597	0.225	1.817	2.652	0.008
Treatment (HL)	–0.237	0.241	0.789	−0.984	0.325
Treatment (HH)	0.225	0.240	1.253	0.941	0.347

### MULTIVARIATE MODELS: ID, G, AND SELECTION THROUGH SURVIVAL

Comparison of unstructured and diagonal models revealed significant among‐trait covariance in **ID**. This was true in pooled‐sex (χ^2^_15_ = 2057, *P* < 0.001), and sex‐specific analyses (males χ^2^_15_ = 1430, *P* < 0.001, females χ^2^_10_ = 663, *P* < 0.001). Length and weight are strongly positively correlated with each other in the pooled sex estimate of **ID** (Table 4), and also with *growth* traits. In other words random intercepts (size at mean age) and slopes (growth) were positively correlated in the random regression models. For males, *dominance score* is positively correlated with all measures of size and growth but, counter to predictions, is negatively associated with *activity*. The correlation between *activity* and *dominance score* is nominally significant based on Z score ≥ 1.96 (*r*_I_ = –0.410 (0.104)).

**Table 4:** Estimates of among‐individual (**ID**) and additive genetic (**G)** matrices

	**TRAIT**	*Activity*	*Dominance score*	*Standard length*	*Weight*	*Growth_SL_*	*Growth_WT_*	*Survival*
**ID**	*Activity*	0.198 (0.029)	**–0.410 (0.104)**	**–0.472 (0.065)**	**–0.513 (0.063)**	**–0.378 (0.069)**	**–0.442 (0.067)**	0.013 (0.074)
	*Dominance score*	–0.084 (0.023)	0.213 (0.032)	**0.632 (0.063)**	**0.711 (0.055)**	**0.493 (0.069)**	**0.649 (0.059)**	0.233 (0.082)
	*Standard length*	–0.010 (0.017)	0.138 (0.020)	0.225 (0.017)	**0.921 (0.009)**	**0.576 (0.037)**	**0.648 (0.033)**	0.189 (0.051)
	*Weight*	–0.123 (0.019)	0.177 (0.022)	0.236 (0.019)	0.292 (0.022)	**0.743 (0.026)**	**0.867 (0.015)**	0.358 (0.048)
	*Growth_SL_*	–5.08 × 10^−4^ (1.05 × 10^−4^)	6.88 × 10^−4^ (1.20 × 10^−4^)	8.25 × 10^−4^ (9.04 × 10^−5^)	1.21 × 10^−3^ (1.10 × 10^−4^)	9.13 × 10^−6^ (7.07 × 10^−7^)	**0.926 (0.008)**	0.391 (0.048)
	*Growth_WT_*	–8.54 × 10^−4^ (1.53 × 10^−4^)	1.30 × 10^−3^ (1.77 × 10^−4^)	1.34 × 10^−3^ (1.34 × 10^−4^)	2.04 × 10^−3^ (1.68 × 10^−4^)	1.22 × 10^−5^ (9.76 × 10^−7^)	1.89 × 10^−5^ (1.47 × 10^−6^)	0.482 (0.044)
	*Survival*	0.005 (0.027)	0.087 (0.032)	0.073 (0.021)	0.157 (0.025)	9.59 × 10^−4^ (1.46 × 10^−4^)	1.70 × 10^−3^ (2.18 × 10^−4^)	0.659 (0.050)
**G**	*Activity*	0.079 (0.049)	**–0.845 (0.361)**	–0.280 (0.344)	**–0.580 (0.287)**	–0.378 (0.323)	**–0.625 (0.286)**	
	*Dominance score*	–0.042 (0.032)	0.032 (0.032)	0.424 (0.422)	**0.795 (0.363)**	0.432 (0.468)	0.736 (0.413)	
	*Standard length*	–0.020 (0.028)	0.0195 (0.023)	0.066 (0.030)	**0.852 (0.093)**	–0.176 (0.330)	–0.080 (0.362)	
	*Weight*	–0.038 (0.029)	0.0334 (0.024)	0.052 (0.028)	0.056 (0.0290)	0.118 (0.346)	0.367 (0.313)	
	*Growth_SL_*	1.95 × 10^−4^ (2.02 × 10^−4^)	1.42 × 10^−4^ (1.76 × 10^−4^)	–8.33 × 10^−5^ (1.52 × 10^−4^)	5.11 × 10^−5^ (1.60 × 10^−4^)	3.39 × 10^−6^ (1.55 × 10^−6^)	**0.890 (0.072)**	
	*Growth_WT_*	3.64 × 10^−4^ (2.67 × 10^‐4^)	2.72 × 10^−4^ (2.24 × 10^−4^)	–4.28 × 10^−5^ (1.89 × 10^−5^)	1.79 × 10^−4^ (2.11 × 10^−4^)	3.40 × 10^−6^ (2.38 × 10^−6^)	4.30 × 10^−6^ (2.38 × 10^−6^)	

Estimates are derived from analyses of both sexes combined (see main text) noting that *Dominance score* is sex‐limited (males only). Among‐individual or additive genetic variances are shown on the diagonal (light grey shading), with between‐trait covariances below the diagonal with corresponding correlations above. Standard errors are shown in parentheses for all estimates and bold font denotes individual correlations deemed nominally significant at α = 0.05 (based on |correlation/SE| ≥ 1.96). The expanded estimate **ID** is shown with *Survival* included as a further response to estimate the selection differentials on each trait (dark grey shading) and corresponding trait‐fitness correlations (black shading).

Noting that *dominance score* is a male limited trait, and excluding relationships with *survival* (see below) the correlation structure in **ID** is qualitatively similar in the two sexes (Table S2) and **G** was thus estimated from a pooled analysis. A diagonal genetic matrix was supported over a null model (χ^2^_6_ = 35.6, *P* < 0.001), and the fully unstructured matrix was significantly better again (χ^2^_15_ = 59.4, *P* < 0.001). The first comparison corroborates the presence of genetic variance, the second provides evidence of among‐trait genetic covariance structure (Table 4). On a correlation scale (i.e., upper diagonal of **G** in Table 4), our estimates of between‐trait genetic relationships largely mirror those in **ID** (although SEs are larger and not all pairwise estimates of r_G_ should be deemed significant). Thus, for example, we find a strong negative genetic correlation between *activity* and *dominance score* (*r*_G_ = –0.845 (0.361)). The former is also negatively genetically correlated with size and growth, for the latter the correlation structure is positive (Table 4).

Finally, expanding the multivariate model used to estimate **ID** to also include relative survival provided evidence for (among‐individual) phenotype‐fitness covariance (both sexes combined χ^2^_6_ = 97.8, *P* < 0.001). Selection differentials, S (contained in the final row of the expanded **ID** matrix; Table 4) and their corresponding correlations indicate positive trait‐fitness associations that are nominally significant for all traits except *activity* (*r*_I_ = 0.013 (0.074)). Thus large, fast growing, dominant (if male) individuals showed higher survival, but *activity* does not predict fitness. In males, the phenotype‐fitness associations were similar (Table S2), and significant (χ^2^_6_ = 104.8, *P* < 0.001). However, single sex analysis provided no statistical support for significant selection on female traits (excluding dominance; χ^2^_5_ = 7.38, *P* = 0.194).

## Discussion

The aims of this study were to ascertain the effects of experimentally manipulated competition on growth, personality, and survival in the sheepshead swordtail, and to investigate among‐individual and genetic covariance structures between traits related to social dominance. We found evidence of reductions in size and growth at high competition as predicted, but no evidence of density effects on personality. Between traits there was significant among‐individual covariance structure, with personality (boldness) predicting social dominance, size, growth, and survival, though not all relationships matched our a priori predictions. Genetic covariance was also found between traits and we detected low, but statistically significant genetic variance in male dominance that has important consequences for the evolution of traits dependent on competitive outcomes. In what follows we first discuss the density effects on phenotype and patterns of phenotypic covariance before addressing these evolutionary implications in more detail.

### THE EFFECTS OF INCREASED COMPETITION ON PHENOTYPE AND FITNESS

As predicted, we found evidence that density (i.e., level of competition for space) influenced phenotypes and fitness. For example, size and growth rates were lower in early life at high density, consistent with the widespread reporting of density‐dependent growth rates in fishes (see e.g., Rothschild 1986; Lorenzen and Enberg 2002; Hixon et al. 2012). Significant density treatment effects on later life growth were also found, and were driven in particular by reduced growth (measured by live mass (WT)) in fish that experienced the low/high (LH) regime. Thus, it seems that switching from a low to a high competition environment part way through development may impose a greater challenge to growth than consistently experiencing high density. Conversely, males experiencing the HL regime actually had the greatest mean size at the end of the experiment. This latter pattern is consistent with compensatory growth, a widely reported phenomenon in fishes entailing a phase of accelerated growth following a period of growth depression, usually when favorable conditions are restored (e.g., Metcalfe and Monaghan 2001; Ali et al. 2003). We also found that survival was directly influenced by the competitive environment. Observed survival was highest in fish experiencing low density throughout life (LL) and lowest in the HH treatment. However, the predictions from survival analysis indicated that, after conditioning on other model effects, the most striking outcome is a significant reduction in survival for fish moved from low to high density (relative to those not moved). This may indicate some form of adaptive plastic response to density in early life, such that individuals raised at low density find themselves maladapted if subjected to an environmental switch. In this case, it is necessarily a plastic within‐generation effect; however, the pattern shows some interesting parallels to results of reciprocal translocation experiments in wild guppies that have been interpreted as evidencing evolution under density‐dependent selection (Bassar et al. 2013).

Reduced allocation to resource dependent traits and a decrease in (absolute) fitness are defining features of competition found ubiquitously across taxa. Thus the reduced growth and survival at higher density are consistent with our density treatment having manipulated the level of competition as intended. We previously reported a weak trend toward later and smaller maturation at high early life density in these fish, highlighting the fact that other aspects of life history are also impacted (Boulton et al. 2016). In contrast however, we found no evidence of density treatment effects on individual behavior. Although this was not unexpected for male dominance (assayed within groups of individuals experiencing the same treatment regime), several recent studies have reported links between density and “bold type” personality variation (see e.g., Patrick et al. 2013; Müller et al. 2016 for observational and experimental studies respectively). Conversely, Niemelä et al. (2012) reported no impact of experimentally manipulated (larval) rearing density on adult boldness in the field cricket *Gryllus integer*, a result that mirrors our lack of population level plastic response of boldness to the density treatment applied.

### AMONG‐INDIVIDUAL CORRELATIONS BETWEEN TRAITS AND FITNESS

After controlling for all fixed effects, our mixed model analyses provided strong evidence of among‐individual variance in those traits with repeated measures (i.e., *activity*, *dominance score*, *size*, and *growth*). Repeatability of *activity*, used here as a proxy for boldness, was known from prior analysis of this data (Boulton et al. 2014). However, consistent among‐male differences in *dominance score* provide independent confirmation that male dominance in this species can be viewed as a repeatable trait of the individual (Wilson et al. 2013), albeit one that will also depend on social context (i.e., group, competitor phenotype). We also found evidence of significant correlations between phenotypic traits (at the among‐individual level) and between traits and fitness, though not all relationships were as predicted. Most strikingly, we had predicted a positive correlation between individual boldness and dominance, but in fact found a strong and highly significant negative one. The strength of the correlation remains consistent with the idea that this personality trait is part of what determines an individual's competitive ability (Briffa et al. 2015), but clearly our directional prediction, based largely on the emerging pattern in the literature (e.g., Dingemanse and de Goede 2004; Sundstrom et al. 2004; Webster et al. 2007; Dahlbom et al. 2011), was entirely wrong. Speculatively, it is possible that the negative association between boldness and dominance reflects alternate male strategies for obtaining resources (food and/or mating opportunities) that have been reported in some *Xiphophorus* species (Ryan and Causey 1989; Zimmerer and Kallman 1989; Ryan and Keddyhector 1992; Cummings and Gelineau‐Kattner 2009). For instance, socially dominant males may be able to hold territories in the natural environment, with subordinates having to use more active, mobile, and exploratory (i.e., bold‐type) behaviors to find undefended resources.

Other correlations in **ID** were more in line with our a priori predictions. Thus, despite being less bold, dominant fish did tend to be larger, and grow faster (as inferred from both standard length and weight). Although *dominance score* is observed for males only, this finding agrees with previous work on the same population where resource acquisition during dyadic interactions was used to assay dominance in both sexes (e.g., Wilson et al. 2013). Under our experimental rearing conditions, viability selection also tends to favor the dominant, faster growing males as predicted. Size and growth were not under significant viability selection in females, although the qualitative pattern of covariance with survival is not dissimilar. Note that following maturity, female fecundity scales tightly with size so we would expect strong (positive) selection on size through lifetime fitness in the wild. We find no evidence of selection on boldness in either sex, but stress that this may well be a consequence of the artificial conditions. For instance, increased predation risk is widely expected to impose a cost on bold behavioral strategies in wild populations. This was recently found in roach (*Rutilus rutilus*), with bolder fish being more susceptible to avian predation (Hulthén et al. 2017). It is also the case that personality traits can be under sexual selection (Schuett et al. 2010), that will not be apparent in our experiment.

### IMPLICATIONS OF GENETIC (CO)VARIANCE

Our animal model analyses confirmed the presence of significant additive genetic effects contributing to observed phenotypic (co)variance. Therefore, there is evidence for genetic variance in boldness (*activity*) and male *dominance score*, as well as in *size* and *growth* (as measured by standard length and weight). The presence of genetic variance means that there is scope for adaptive evolution (Falconer and Mackay 1996) although the extent that the traits involved can respond independently to selection on them will depend on the genetic covariance/correlation structure in **G** (Walsh and Blows 2009). Overall, there was statistical support for between‐trait genetic covariance although we acknowledge that pairwise genetic correlations between traits were characterised by high levels of uncertainty. Nonetheless, a number of correlations were nominally significant at α = 0.05 (based on their estimated standard errors). This included, for example, the strong negative estimate of r_G_ between boldness and male dominance. In this case, and more generally, the sign of the estimated genetic correlations matched that of the phenotypic correlations in **I** as discussed above.

Two results from our genetic analysis are worth highlighting. The first is that the among‐individual variation in boldness previously reported (Boulton et al. 2014), is underpinned by significant heritable variation. Although it has long been known that genes influence personality in humans (e.g., Horn et al. 1976; Jang et al. 1996; Bouchard and McGue 2003) comparable studies on animals, particularly wild ones, are still quite rare (but see: Drent et al. 2003; Dingemanse et al. 2004; van Oers et al. 2004). Our result thus adds to an emerging picture of genetic differences among individuals being important determinants of animal personality generally (Dochtermann et al. 2015) and in fishes specifically (Dingemanse et al. 2012).

A second important result, and one more germane to our study rationale, is that we found evidence for heritable variation in male *dominance score*. Although the estimate of h^2^ is low (6.6%), genetic correlations with other traits examined suggest that this could have important evolutionary consequences. This is because if the resources won by a focal individual in competition depend on its own genotype, it follows that they will also be influenced by the genotypes of competitors, giving rise to indirect genetic effects (IGEs; Moore et al. 1997; Moore et al. 2002) on resource acquisition and resource dependent traits. While IGE can accelerate selection responses in some contexts (Wolf et al. 1998), under competition they are expected to constrain phenotypic responses of resource‐dependent traits (e.g., size, growth) to directional selection as a result of the evolution of a more competitive social environment (Hadfield 2010; see Introduction). Equivalently, but conceptualized slightly differently, IGEs reduce the genetic variance available to facilitate a selection response (Wilson 2014). In the limiting case with a finite resource and constant population size, a response to selection in a single trait will depend not on the total magnitude of V_A_ but on the portion that is independent of competitive ability (and thus free from constraining IGE; Wilson 2014). If *dominance score* is a valid measure of competitive ability, then from our estimate of **G** we can determine this as V_A|Dom_/V_A_ where V_A|Dom_ is the additive variance conditional on *dominance score*. Following Hansen and Houle 2008, for a single trait y conditional on a single trait x we can calculate V_Ay|x_ = V_A(y)_ ‐ COV_A(y,x)_·V_A(x)_^−1^ ·COV_A(x,y)._ This yields, for example, values of V_A|Dom_/V_A_ of 38% and 42% respectively for size and growth as measured by live weight.

The presence of heritable variation for dominance coupled to positive genetic covariance with size and growth, thus implies that IGEs arising from competition could reduce observed selection responses by >50% relative to naive predictions. Two caveats need stating however. First this degree of constraint is likely to be an overestimate because it assumes that size and growth are causally dependent on competitive ability but not *vice versa*. We have no way to assess causality from our data although there are a wealth of contest studies in *Xiphophorus* showing that body size is a key predictor of contest outcome (see Earley and Hsu 2008 for a review). Thus the (genetic) covariances between dominance and size and growth are likely to reflect bidirectional causality. Nonetheless, we have previously shown that the presence of dominant *X. birchmanni* males reduces weight gain of subordinate tank mates (Wilson et al. 2013). The specific mechanism underpinning this is unknown. Reduced growth rates in behaviorally subordinate fish could be an indirect consequence of experiencing harassment and bullying from fish with dominant phenotypes (as opposed to a direct consequence of obtaining less resource, e.g., food). It is well known that physiological effects of chronic social stressors such as bullying can impact behavior, health, life history, and survival in animal populations (e.g., Pickering and Pottinger 1989; Boonstra et al. 2001; Barton 2002). Individual fitness may depend therefore not only on the ability to win resources (and thus the phenotypic traits that promote resource winning) but also on the ability to cope with the social stress imposed by socially dominant conspecifics.

A second caveat to note is that we did not explicitly model or estimate IGEs on traits presumed consequent to competitive outcomes (i.e., size, growth). Ideally this would have allowed us to verify the expected consequences of heritable dominance for downstream traits (as discussed above). Although in principle it is relatively straightforward to estimate IGEs within a variance partitioning animal model framework (Bijma et al. 2007), we were unable to obtain stable model convergence from our data. We acknowledge that our breeding design is relatively small here and therefore data availability is likely limiting in this regard (as well as contributing to high uncertainty in the elements of **G**). In fact, a second generation of breeding was initially planned to allow further investigation of IGEs_,_ as well as testing for G x E across the density treatments. However, this was precluded by poor reproductive success of surviving fish both during and following the current experiment. We therefore note that more complex quantitative genetic models are thus unlikely to provide further insight.

## Conclusions

In summary, this study sought to investigate the direct effects of social competition on phenotype and fitness, test for among‐individual variation in competitive ability (i.e., dominance) and investigate the multivariate genetic architecture linking traits putatively causal and consequent to dominance. We found that higher levels of competition caused reductions in growth and survival but had no effect on average behavior. *Dominance score* was repeatable in males, and positively correlated with size, growth, and survival at the among‐individual level as predicted. However, while we found a correlation between personality (boldness) and dominance, the sign of this relationship was negative counter to our predictions. Thus, fish that were bolder actually tended to be less dominant (if male). This is compatible with the premise that personality is an important determinant of social dominance, but the direction of the relationship is something of an anomaly when set against the wider context of empirical studies of boldness. We also found evidence of genetic (co)variance underpinning observed phenotypic variation. Thus there is genetic integration between boldness, dominance, size, and growth, and these aspects of phenotype will not evolve independently under selection. Of particular significance is the conclusion that dominance is both heritable and genetically correlated with size and growth. Provided growth depends on the outcome of competition, heritable dominance leads to an expectation of indirect genetic effects that will act as constraints on selection responses.

Associate Editor: T. Flatt

Handling Editor: M. Noor

## Supporting information


**Table S1**. Estimated coefficients from univariate animal models of each trait.
**Table S2**. Estimates among‐individual (**ID**) matrices for males (**ID_M_**) and females (**ID_F_**) separately.
**Figure S1**. Observed size (A ‐ standard length, B ‐ live mass) and relative growth (C,D) by age for female (red), male (green) and fish of unknown sex (blue).
