# Supplementary material for: Phenotypic and genetic integration of personality and growth under competition in the sheepshead swordtail, Xiphophorus birchmanni
Source: Evolution. 2017 Nov 30;72(1):187–201. doi: 10.1111/evo.13398 (PMC5814916; doi:10.1111/evo.13398)
Supplement: Supplementary file 1 — Table S1. Estimated coefficients from univariate animal models of each trait. Table S2. Estimates among‐individual (ID) matrices for males (IDM) and females (IDF) separately. Figure S1. Observed size (A ‐ standard length, B ‐ live mass) and relative growth (C,D) by age for female (red), male (green) and fish of unknown sex (blue). [file EVO-72-187-s001.pdf]

Supplemental Materials

Supplemental table 1: Estimated coefficients from univariate animal models of each trait. Standard errors are shown in parentheses (but not for reference levels of the factors that have an effect size of zero). See main text for statistical inference and details of units for traits and predictor variables. Note also that age-dependence of size traits (standard length and weight) is a function of both *Age* and *Measure* (and interactions involving the latter).

**Trait**	**Effect** (factor level)	**Coefficient** (SE)
Activity	Order	-0.131 (0.041)
	Trial (1)	0 (-)
	Trial (2)	16.0 (1.05)
	Trial (3)	18.5 (1.11)
	Trial (4)	18.0 (1.22)
	Sex (M)	-3.32 (1.21)
	Stack (A)	0 (-)
	Stack (B)	1.29 (2.30)
	Stack (D)	9.07 (2.25)
	Stack (E)	10.2 (2.29)
	Stack (F)	6.10 (2.35)
	Stack (G)	0.411 (2.24)
	Intercept	62.1 (2.55)
Dominance score	Trial (1)	0 (-)
	Trial (2)	-0.453 (0.981)
	Trial (3)	-0.899 (1.00)
	Trial (4)	-1.65 (1.10)
	Trial (5)	-0.080 (1.25)
	Trial (6)	1.42 (1.35)
	Trial (7)	2.81 (1.35)
	Trial (8)	2.60 (1.35)
	Trial (9)	3.12 (1.36)
	Trial (10)	5.95 (1.37)
	Trial (11)	3.09 (1.86)
	Trial (12)	2.49 (1.86)
	Trial (13)	-0.536 (1.86)
	Trial (14)	1.71 (1.88)
	Trial (15)	2.57 (2.19)
	Trial (16)	4.49 (3.27)
	Intercept	2.24 (0.956)
Standard length	ELD_{Part1} (H)	0 (-)
	ELD_{Part1} (L)	0.635 (0.113)
	Measure:Sex (1:Male)	0 (-)
	Measure:Sex (2:Male)	0.211 (0.154)
	Measure:Sex (3:Male)	0.306 (0.175)
	Measure:Sex (4:Male)	-0.037 (0.205)
	Measure:Sex (5:Male)	-0.258 (0.240)

	Measure:Sex (6:Male)	-0.622 (0.281)
	Measure:Sex (7:Male)	-1.04 (0.323)
	Measure:Sex (8:Male)	-1.69 (0.369)
	Measure:Sex (9:Male)	-1.96 (0.412)
	Measure:Sex (10:Male)	-2.48 (0.464)
	Measure:Sex (11:Male)	-3.00 (0.512)
	Measure:Sex (12:Male)	-3.65 (0.563)
	Measure:Sex (13:Male)	-4.28 (0.607)
	GS	0.337 (0.126)
	Sex (M)	-0.275 (0.267)
	Stack (A)	0 (-)
	Stack (B)	0.975 (0.668)
	Stack (D)	1.44 (0.648)
	Stack (E)	0.704 (0.672)
	Stack (F)	2.20 (0.802)
	Stack (G)	1.10 (0.744)
	Measure (1)	0 (-)
	Measure (2)	2.00 (0.375)
	Measure (3)	3.37 (0.612)
	Measure (4)	4.98 (0.866)
	Measure (5)	5.51 (1.13)
	Measure (6)	6.16 (1.40)
	Measure (7)	6.24 (1.67)
	Measure (8)	6.52 (1.94)
	Measure (9)	6.81 (2.21)
	Measure (10)	6.80 (2.51)
	Measure (11)	6.91 (2.78)
	Measure (12)	7.18 (3.06)
	Measure (13)	7.36 (3.31)
	Age	0.046 (0.010)
	Intercept	32.98 (1.41)
Weight	$(LLD:ELD)_{Part2}$ (H:H)	0 (-)
	$(LLD:ELD)_{Part2}$ (H:L)	0 (-)
	$(LLD:ELD)_{Part2}$ (L:H)	-0.165 (0.04)
	$(LLD:ELD)_{Part2}$ (L:L)	-0.061 (0.038)
	LLD_{Part2} (H)	0 (-)
	LLD_{Part2} (L)	0.024 (0.033)
	ELD_{Part1} (H)	0 (-)
	ELD_{Part1} (L)	0.050 (0.020)
	Measure:Sex (1:Male)	0 (-)
	Measure:Sex (2:Male)	0.014 (0.027)
	Measure:Sex (3:Male)	0.030 (0.030)
	Measure:Sex (4:Male)	-0.011 (0.034)
	Measure:Sex (5:Male)	-0.052 (0.039)
	Measure:Sex (6:Male)	-0.097 (0.045)
	Measure:Sex (7:Male)	-0.160 (0.052)

Measure:Sex (8:Male)	-0.242 (0.059)
Measure:Sex (9:Male)	-0.315 (0.065)
Measure:Sex (10:Male)	-0.38 (0.073)
Measure:Sex (11:Male)	-0.490 (0.081)
Measure:Sex (12:Male)	-0.618 (0.089)
Measure:Sex (13:Male)	-0.712 (0.096)
Sex (M)	-0.013 (0.027)
Stack (A)	0 (-)
Stack (B)	0.120 (0.060)
Stack (D)	0.184 (0.058)
Stack (E)	0.106 (0.060)
Stack (F)	0.213 (0.073)
Stack (G)	0.077 (0.068)
Measure (1)	0 (-)
Measure (2)	0.108 (0.053)
Measure (3)	0.188 (0.073)
Measure (4)	0.345 (0.097)
Measure (5)	0.450 (0.123)
Measure (6)	0.516 (0.150)
Measure (7)	0.540 (0.178)
Measure (8)	0.593 (0.205)
Measure (9)	0.717 (0.233)
Measure (10)	0.728 (0.264)
Measure (11)	0.823 (0.291)
Measure (12)	0.990 (0.321)
Measure (13)	0.974 (0.346)
Age	0.004 (0.001)
Intercept	1.05 (0.141)

Supplemental Table 2: Estimates among-individual (**ID**) matrices for males (**ID$_M$**) and females (**ID$_F$**) separately. Among-individual variances are shown on the shaded diagonal, with between-trait covariances below and correlations above. Standard errors are shown in parentheses and bold font denotes individual off diagonal elements that are nominally significant at α = 0.05 (based on |estimate/SE| ≥ 1.96). *Dominance scores* available for males only. Directly observed traits (*Activity, Dominance score, Standard length and Weight*) were scaled to standard deviation units prior to modelling. *Survival* was mean standardised for inclusion in the expanded **ID** matrix estimates.

		Activity	*Dominance score*	*Standard length*	*Weight*	*Growth$_{SL}$*	*Growth$_{WT}$*	*Survival*
**ID$_M$**	*Activity*	0.205 (0.040)	**-0.461 (0.108)**	**-0.569 (0.078)**	**-0.578 (0.078)**	**-0.407 (0.088)**	**-0.463 (0.086)**	-3.00x10^{-4} (0.098)
	Dominance score	-0.099 (0.027)	0.226 (0.034)	**0.644 (0.063)**	**0.717 (0.056)**	**0.558 (0.069)**	**0.677 (0.059)**	**0.333 (0.083)**
	Standard length	-0.131 (0.024)	0.156 (0.024)	0.257 (0.026)	**0.934 (0.010)**	**0.685 (0.038)**	**0.717 (0.036)**	**0.308 (0.064)**
	Weight	-0.159 (0.029)	0.207 (0.029)	0.288 (0.030)	0.370 (0.037)	**0.818 (0.025)**	**0.901 (0.015)**	**0.492 (0.054)**
	Growth$_{SL}$	-6.25x10^{-4} (1.56x10^{-4})	8.99x10^{-4} (1.57x10^{-4})	1.18x10^{-3} (1.46x10^{-4})	1.68x10^{-3} (1.85x10^{-4})	1.15x10^{-5} (1.15x10^{-6})	**0.942 (0.009)**	**0.493 (0.055)**
	Growth$_{WT}$	-1.02x10^{-4} (2.28x10^{-4})	1.57x10^{-3} (2.33x10^{-4})	1.77x10^{-3} (2.13x10^{-4})	2.66x10^{-3} (2.77x10^{-4})	1.55x10^{-5} (1.59x10^{-6})	2.37x10^{-5} (2.36x10^{-6})	**0.608 (0.047)**
	Survival	-1.05x10^{-4} (0.039)	0.138 (0.039)	0.136 (0.033)	0.260 (0.042)	1.45x10^{-3} (2.35x10^{-4})	2.57x10^{-3} (3.54x10^{-4})	0.757 (0.076)
**ID$_F$**	*Activity*	0.192 (0.043)	-	-0.216 (0.112)	**-0.31 (0.111)**	**-0.298 (0.116)**	**-0.361 (0.111)**	-0.027 (0.118)
	Dominance score	-	-	-	-	-	-	-
	Standard length	-0.034 (0.019)	-	0.131 (0.016)	**0.920 (0.015)**	0.163 (0.090)	**0.466 (0.073)**	0.074 (0.087)
	Weight	-0.057 (0.022)	-	0.139 (0.018)	0.174 (0.022)	**0.442 (0.075)**	**0.747 (0.044)**	0.145 (0.088)
	Growth$_{SL}$	-3.07x10^{-4} (1.29x10^{-4})	-	1.39x10^{-4} (7.90x10^{-5})	4.32x10^{-4} (9.68x10^{-5})	5.51x10^{-6} (6.98x10^{-7})	**0.899 (0.018)**	**0.188 (0.093)**
	Growth$_{WT}$	-5.78x10^{-4}	-	6.07x10^{-4} (1.29x10^{-4})	1.12x10^{-3} (1.66x10^{-4})	7.59x10^{-6} (1.02x10^{-6})	1.30x10^{-5} (1.65x10^{-6})	**0.241 (0.090)**
	Survival	-0.009 (0.039)	-	0.020 (0.024)	0.045 (0.0284)	3.29x10^{-4} (1.70x10^{-4})	6.47x10^{-4} (2.61x10^{-4})	0.558 (0.067)

Supplemental Figure 1: Observed size (A - standard length, B - live mass) and relative growth (C,D) by age for female (red), male (green) and fish of unknown sex (blue). Each point represents a single observation and lines show smoothed fits through the data (pooled across treatments) for illustrative purposes only. Note the data contain repeat records and no statistical inference is intended. Relative growth rate at time *t* was defined as $100*\ln(size_t/size_{t\text{-}1month})$ with size measured as either standard length (C) or live mass (D). Dotted and dashed vertical lines indicate average age of maturity in males (205 days) and females (228 days) respectively, with maturity status inferred from external morphology (see Boulton et al. 2016 for details).

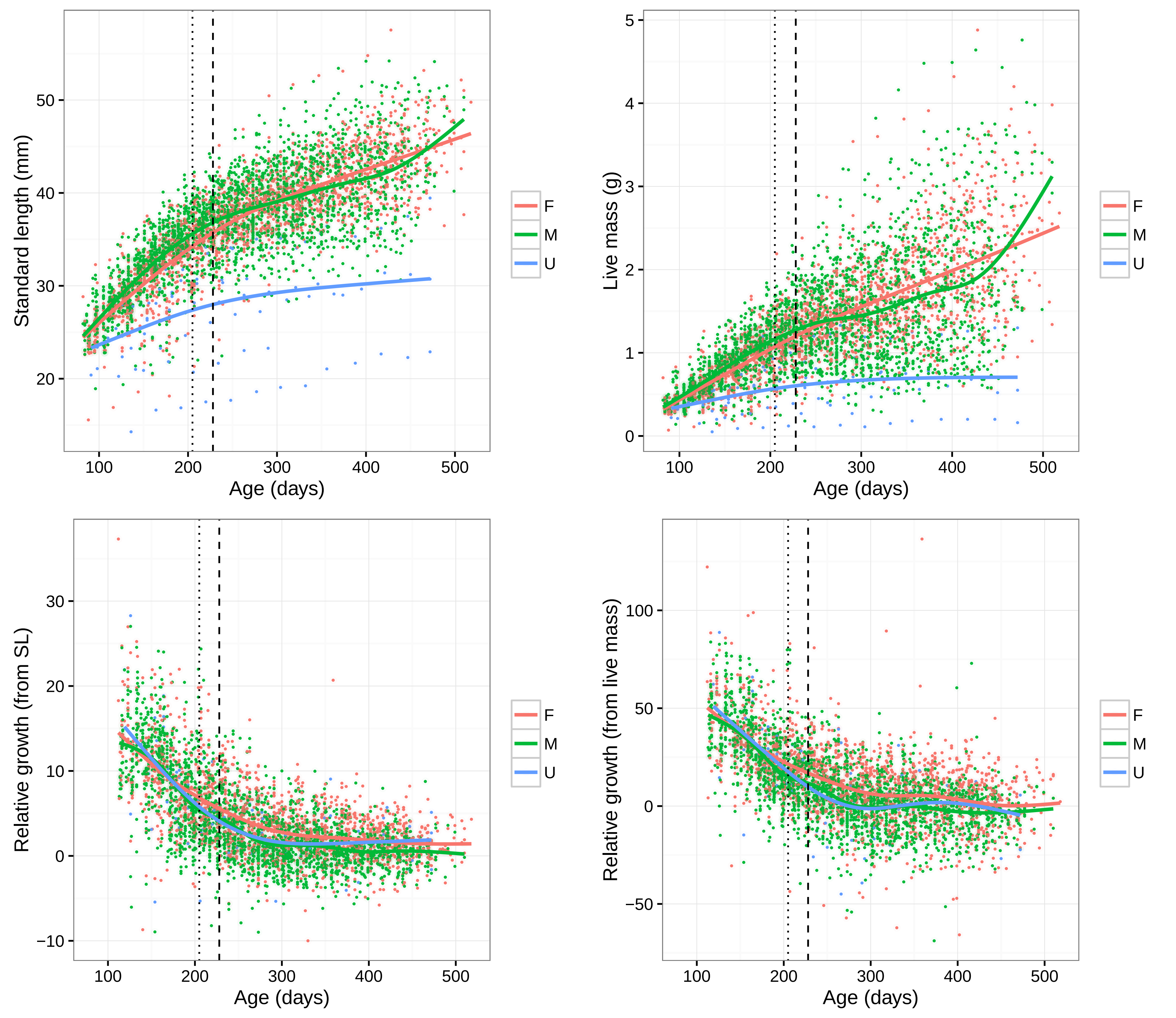

Standard length (mm)
Age (days)
Live mass (g)
Relative growth (from SL)
Relative growth (from live mass)
F
M
U
